# Beyond signal functions in global obstetric care: Using a clinical cascade to measure emergency obstetric readiness

**DOI:** 10.1371/journal.pone.0184252

**Published:** 2018-02-23

**Authors:** John N. Cranmer, Julia Dettinger, Kimberly Calkins, Minnie Kibore, Onesmus Gachuno, Dilys Walker

**Affiliations:** 1 Emory University, Atlanta, Georgia, United States of America; 2 University of Washington, Department of Global Health, Seattle, Washington, United States of America; 3 University of Nairobi, Department of Paediatrics & Child Health Lecturer, Kenyatta National Hospital, Nairobi, Kenya; 4 University of Nairobi, Department of Obstetrics & Gyneacology, Kenyatta National Hospital, Nairobi, Kenya; 5 University of California—San Francisco School of Medicine, Department of Obstetrics, Gynecology and Reproductive Sciences, San Francisco, California, United States of America; Stellenbosch University, SOUTH AFRICA

## Abstract

**Background:**

Globally, the rate of reduction in delivery-associated maternal and perinatal mortality has been slow compared to improvements in post-delivery mortality in children under five. Improving clinical readiness for basic obstetric emergencies is crucial for reducing facility-based maternal deaths. Emergency readiness is commonly assessed using tracers derived from the maternal signal functions model.

**Objective-method:**

We compare emergency readiness using the signal functions model and a novel clinical cascade. The cascades model readiness as the proportion of facilities with resources to *identify* the emergency (stage 1), *treat* it (stage 2) and *monitor-modify* therapy (stage 3). Data were collected from 44 Kenyan clinics as part of an implementation trial.

**Findings:**

Although most facilities (77.0%) stock maternal signal function tracer drugs, far fewer have resources to practically identify and treat emergencies. In hypertensive emergencies for example, 38.6% of facilities have resources to identify the emergency (Stage 1 readiness, including sphygmomanometer, stethoscope, urine collection device, protein test). 6.8% have the resources to *treat* the emergency (Stage 2, consumables (IV Kit, fluids), durable goods (IV pole) and drugs (magnesium sulfate and hydralazine). No facilities could *monitor* or *modify* therapy (Stage 3). Across five maternal emergencies, the signal functions overestimate readiness by 54.5%. A consistent, step-wise pattern of readiness loss across signal functions and care stage emerged and was profoundly consistent at 33.0%.

**Significance:**

Comparing estimates from the maternal signal functions and cascades illustrates four themes. First, signal functions overestimate practical readiness by 55%. Second, the cascade’s intuitive indicators can support cross-sector health system or program planners to more precisely measure and improve emergency care. Third, adding few variables to existing readiness inventories permits step-wise modeling of readiness loss and can inform more precise interventions. Fourth, the novel aggregate readiness loss indicator provides an innovative and intuitive approach for modeling health system emergency readiness. Additional testing in diverse contexts is warranted.

## Introduction

Protracted elevations in global labor-related deaths persist despite 71% of births now being attended by skilled professionals [1–3]. Between 1990 and 2015, global labor-related deaths declined much more slowly compared to post-partum deaths. Further, the under-five mortality ratio shrunk by 2.12% annually during this period while maternal mortality reductions were much slower at 1.80% [1] and perinatal mortality reductions were essentially stagnant [4–10]. The majority of peripartum deaths are driven by labor-related disorders including maternal hemorrhage, eclampsia and maternal/perinatal sepsis [4, 7, 11, 12]. Given this protracted facility-based mortality, mobilizing clinical resources for labor-related emergency care may be a crucial step for reducing persistent facility-based mortality [13–15]. Mobilizing and distributing such resources is based on accurate measurement of emergency readiness.

In 1993, the World Health Organization (WHO) identified essential resources for managing common obstetric emergencies [16]. The WHO’s Mother-Baby Package codified these resources into eight facility-based actions to treat the major causes of global maternal death at facilities [17]. This pioneering approach evolved into the signal functions framework and emergency obstetric care (EOC) [18–23]. The basic maternal signal functions include six clinical actions used during obstetric emergencies; three are medical treatment signal functions and three are manual procedure functions [22]. The three medical signal functions involve administration of: parenteral antibiotics, parenteral anticonvulsants and parenteral oxytocics. The three manual procedure functions are: assisted vaginal delivery, removal of retained products of conception and removal of retained placenta.

Specific items—or tracers—used for each signal function have been used as proxy measures for overall emergency readiness—or a facility’s ability to manage obstetric emergencies [23–29]. This signal function method has become the dominant approach for measuring global readiness at facilities [30–32]. The WHO created a Service Availability and Readiness Assessment tool (SARA) used to summarize the readiness—or resource availability—for a broad range of clinical services [33]. The obstetric-specific Service Readiness Index (SRI) defines a facility’s aggregate obstetric emergency readiness using the mean number of 11 tracers present on the day of observation at the facilities [32, 33]. This WHO method has been applied in multiple global contexts [30, 31, 33].

There has been growing consensus, however, on the need to revise or modify readiness assessments derived from the signal functions [25, 27, 34–38]. To our knowledge, signal functions, tracer items and readiness scores have not yet been used to predict a facility’s ability to manage specific emergencies or to predict labor-related health outcomes [34, 36]. Consequently, to substantively reduce delivery-related mortality on a global scale, additional work is needed to define intuitive, relevant, measurable indicators [39] that simultaneously predict survival [37] and are simultaneously relevant for clinics and health systems.

The capacity hierarchy of needs model identifies a predictable, interdependent relationship between health systems, facilities, clinician skill and the tools clinicians use to provide clinical care [40]. Clinical tools—such as drugs—can only be utilized when staff posses the tools, skills, knowledge and infrastructure required to administer those drugs. In health care systems, a predictable cascade of patient loss has been documented between initial diagnosis and sustained treatment for HIV and other diseases [41, 42].

### Study rationale, research question and context

As part of a baseline facility inventory of a larger obstetric quality improvement implementation trial in Kakamega County, Kenya, we designed a nested descriptive study to 1) measure the signal functions ability to practically describe a facility’s clinical readiness to manage basic obstetric emergencies and 2) test a novel emergency obstetric readiness model. In Kenya, the *ratio* of pregnant women dying as a consequence of pregnancy has remained stable for over 30 years (maternal mortality ratio = 400 (MMR)) [43, 44]. Among all nations, Kenya bears the eighth highest total number of deaths [43, 45, 46]. A recent service readiness inventory suggests elevations in mortality may be partially attributed to limited clinical resources for managing emergencies. Across 7,995 facilities, Kenya’s preparedness for managing obstetric emergencies (as measured by the proportion of facilities with specified signal function resources) was 32%. Similar trends were present among public facilities (32%) and in Kakamega County (38%) [31]. One region in Kakamega County was used to test the proposed obstetric emergency clinical cascades at Ministry of Health (MoH) facilities and to compare the new model’s performance with readiness estimates from the standard signal functions model.

## Materials and methods

### Setting

Kakamega County was selected by the Government of Kenya for a parent implementation trial testing the impact of a package of community- and clinical quality interventions on the uptake and quality of facility-based care. Kakamega was selected by the MoH based on government data indicating the county’s MMR of 800 was nearly double the country’s ratio of 413 [44, 47]. Forty four facilities and their catchment areas were selected for intervention using four criteria: primary care clinics with KEPH Level 2–3 designation (Kenya Essential Package of Health [47]), providing basic emergency obstetric services as defined by the MoH (BEmOC) [48], conducting 10 or more deliveries in the previous calendar year (2011) and being located in one of five sub-counties within Kakamega County (for the purpose of analysis, two facilities that were formerly part of Kakamega Central prior to post-constitutional rezoning were retained in the study based on the intent-to-treat principle. Thus, the results report on five sub-counties since the newly designated Navakholo county was retained with Kakamega Central for the parent trial.

### Study design

The cross-sectional analysis of facility readiness is nested within a non-equivalent group design pre-post implementation trial evaluating a facility- and community-intervention package in Kakamega County, Kenya [49]. In the parent trial, 756 facility-specific variables were collected at 44 primary care facilities; this nested study used 80 obstetric-specific variables collected from facilities prior to the start of the intervention.

### Emergency readiness

Obstetric emergency readiness at the facility-level has been defined by the proportion of specified clinical items that are present at a facility on the day a facility inventory is conducted [33]. Although there is no universal consensus on the number of tracers that should be used to measure emergency readiness as defined by the signal functions model [30–33, 50], WHO’s Service Readiness Index (SRI) defines basic emergency obstetric readiness using 7 tracers (composed of 9 discrete items). These tracers are measured using observation and/or interview during facility visits [33]. This signal function-based approach to estimating emergency readiness uses 3 parenteral drugs (uterotonic, antibiotics, anticonvulsant), 3 intravenous items (including IV solution and a 2-part IV infusion kit), 1 durable good (manual vacuum apparatus) and 2 multipurpose items (gloves and light source).

The WHO-SRI standardized tool was used to create a signal function-based estimate of emergency readiness at the 44 primary care clinics. Next, we measured readiness using a novel emergency obstetric clinical cascade model derived from Potter’s hierarchy of needs framework [40] and the HIV care cascade model [42]. The resulting obstetric clinical cascade quantifies resources required to sequentially identify, treat and manage basic obstetric emergencies as they present clinically at primary care facilities. Consequently, emergency obstetric readiness is reported as the percentage of facilities with all of the related clinical tools for managing obstetric emergencies (as defined by the two models). The higher the percentage is, the higher the facilities’ readiness is to manage basic obstetric emergencies. Although facility-level estimates of readiness could be calculated, this study reports the percent readiness aggregated across all 44 clinics.

### Emergency readiness using signal functions

The current signal function readiness estimates are reported for all basic maternal emergency obstetric signal functions as a single indicator—the proportion of facilities with tracer items for all manual procedures and all medical treatments. Standard signal function estimates and the WHO’s obstetric service readiness index (SRI) do not measure readiness for each clinical disorder. However, if one estimated emergency-specific readiness using the signal function tracer items alone, many resources required to practically deliver care would be absent. For example, signal function estimates for eclampsia would be defined as the proportion of facilities with IV solution/infusion set, hydralazine and magnesium sulfate [33]. Using these three items to model eclampsia emergency readiness alone does not account for the resources required to first identify if the emergency is present (i.e., sphygmomanometer, stethoscope, urine collection device and urine protein test). Also, it does not explicitly model all necessary drugs or ancillary resources required to practically deliver the first-line treatment. Although consumable supplies are required to deliver treatment drugs, consumable resources are often omitted from the signal functions for most emergencies (for example, the required refrigeration for oxytocin is not modeled in the oxytocic function and IV tubing, IV catheter and IV solution are discrete, interdependent items that are only measured as one item in signal function estimates. Consequently, reporting capacity when one or more of the three interdependent items are missing is not precise or accurate). Further, although durable goods are essential for conducting procedures or delivering drugs, they are often excluded from signal function estimates (i.e., IV poles, syringes or needles for delivering drugs). For some emergencies, the specific drug required for the emergency is not modeled by the signal functions (For example, in the oxytocic signal function, the cause of post-partum hemorrhage (PPH) and the varied drugs for treating hemorrhage based on its underlying cause are not modeled).

### Emergency readiness using cascades

**Fig 1:**
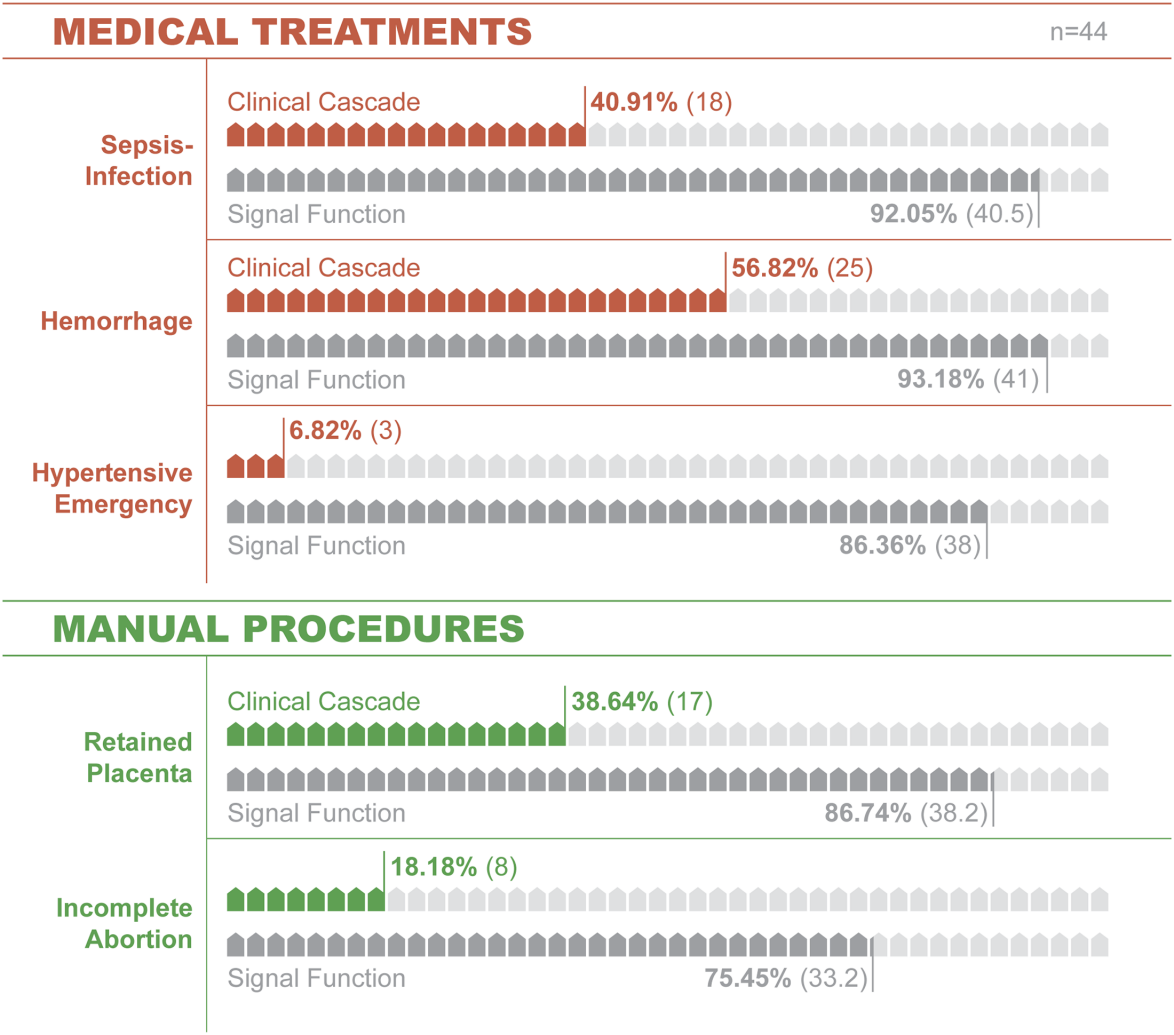
Signal function versus clinical cascade estimates of emergency readiness.

In contrast, the proposed cascade model is a clinically-oriented approach to measuring readiness. It is based on a practical, step-wise cascading relationship between resources [40, 42]. The resources for identifying the emergency (Stage 1) are required first before accurate treatments can be administered to patients (Stage 2). Further, the cascade explicitly models the consumable supplies and durable goods required to practically deliver treatment drugs in clinical practice (for example, in eclampsia, the cascades model the interrelationship between all clinical resources required to first identify the disorder and then deliver the treatment drug). Thus, emergency readiness in the cascade model is the proportion of facilities with the treatment drug that can first identify the disorder (stage 1, Identify) and then have the durable and consumable resources to administer the treatment drug (stage 2, Treat). Since the signal functions do not measure care quality, the third cascade stage for monitoring and modifying therapy as clinically indicated (Stage 3) is not used to compare the signal functions and cascade models (Fig 1).

### Clinical logic of cascade readiness estimates

**Fig 2:**
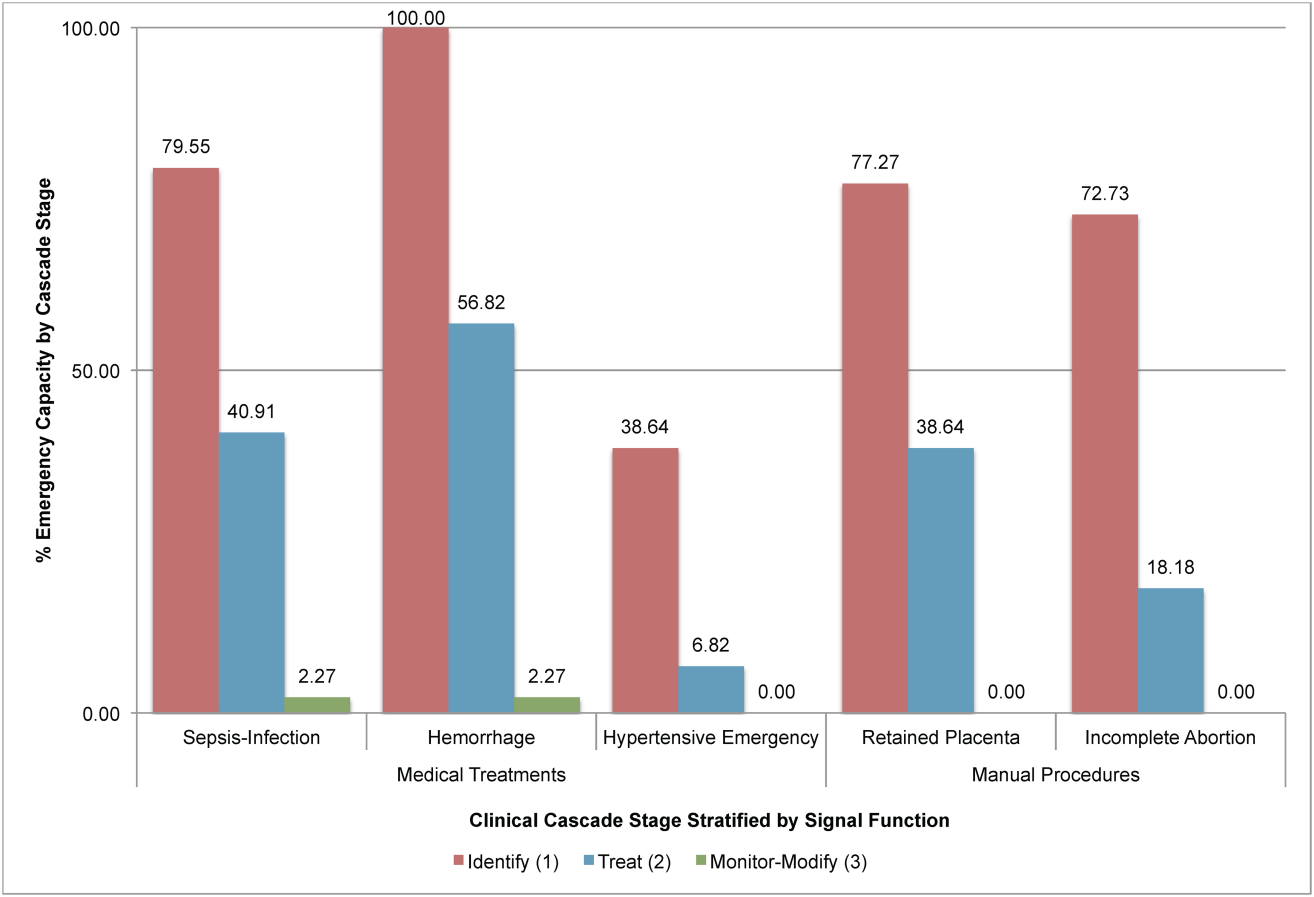
Emergency readiness estimates by emergency cascade and stage.

Each emergency cascade’s title is based on the underlying clinical disorder and paired with existing signal functions as follows: Manage Sepsis-Infection (the parallel signal function is parenteral antibiotics), Hemorrhage (oxytocics), Hypertensive Emergency (anticonvulsant), Retained Placenta (manual removal of retained placenta), Incomplete Abortion (removal of retained products of conception) [22]. A facility’s ability to monitor or modify the primary treatment based on a patient’s clinical response (Stage 3) is a proposed indicator for measuring clinical quality but not for evaluating signal function performance (Fig 2).

### Operational definitions for ambiguous signal function tracers

Tracers were precisely defined to minimize ambiguity in signal function estimates and to facilitate comparison between models. When tracers were not explicitly defined by the signal functions, the WHO first-line recommendations for obstetric care were used [23, 51, 52] to make a general tracer from the signal function model (i.e., parenteral antibiotics) more specific (i.e., parenteral ampicillin and/or penicillin alternative). Four other ambiguous tracers included light source, IV supplies, drugs and emergency protocols. Light was defined as functional electric lights or functional flashlights. The IV kits tracer includes three discrete resources: drug-compatible fluids, tubing and a venous access device/cannula. We modeled this using the cannula and fluids since data on tubing were absent. Fluid was modeled as Lactated Ringer’s/Hartman solution or normal saline since both fluids are compatible with emergency drugs available at the study facilities.

For the cascades, the first-line clinically-indicated drug was used to model readiness when the drug was not defined by the signal function model. The oxytocics signal function does not specify the precise utertonic drug required for various hemorrhage emergencies. When managing obstetric hemorrhage, the preferred first-line drug is oxytocin [23, 51]. However, in its absence, misoprostol or ergometrine could be substituted (with blood pressure monitoring). In contrast, when managing incomplete abortion, the preferred uterotonic is a non-oxytocin agent such as ergometrine or misoprostol. Therefore, the presence of *any first- or second-line uterotonic specific to the emergency* was used to model readiness in the cascade model since the cascades are based on specific clinical emergencies [23, 51].

Further, the parentral antibiotic signal function does not define the tracer antibiotic drugs required. We used the WHO’s 3-step sequence of obstetric antibiotic therapy escalation based on the type of suspected infection to define readiness: Step 1—ampicillin, 2—gentamicin and 3—metronidazole [51]. Since most facilities lack ampicillin, the presence of ampicillin or any of three alternative penicillin drugs (benzathine, procaine or crystalline) was used to model this WHO step 1 antibiotic readiness (the primary study’s clinical inventory did not capture metronidazole availability, so WHO’s step 3 antibiotic readiness was not modeled).

### Emergency readiness and clinical quality

Although some emergency protocols are tracers in the signal function model, they were selectively used to model the quality of clinical care but not general emergency readiness for four reasons: 1) individual clinician knowledge and skill vary so having a protocol does not guarantee readiness, 2) by extension, protocol absence does not guarantee a lack of emergency readiness, 3) signal functions do not define the protocol required [33] and 4) several protocols may actually be required to manage the primary emergency’s sequella. For example, when a patient presents with a retained placenta, a subset of patients may develop post-partum hemorrhage (PPH), endometritis and/or sepsis while another subset may resolve with first-line treatment.

### Study assumptions

Identifying some clinical disorders is based primarily on clinician skill. Although clinician skills vary widely [53], a 100% skill level was assumed for all cascades since skill assessment is not include the signal function estimates of facility emergency readiness [22, 33]. Some items required for comprehensively modeling readiness in all six maternal signal functions were absent from the baseline facility inventory. This analysis focuses on three basic medical and two manual functions since data on assisted vaginal delivery supplies were absent [21, 22]. However, the expanded cascades in supplemental tables include resources for all six maternal cascades.

### Data collection

In parallel with the WHO- readiness estimates based on the signal functions, this study measured the cross-sectional availability of routine and emergency obstetric resources during facility visits conducted by study staff between February and May 2013. Three trained research assistants used standardized forms to visually identify emergency resource availability and ask clinic mangers about resource availability when items were not initially located using observation. This method of survey data collection matches the WHO-SRI approach used to quantify signal function estimates of emergency readiness [33]. 80 variables from the inventory describe facility demographics, staff, consumable medical supplies, durable goods and obstetric drugs. Mean estimates of maternal signal function readiness are derived from 396 observations (9 tracer items from 44 facilities). Cascade estimates of readiness utilized 1,364 observations (31 variables from 44 facilities).

One author (JD) trained all staff on this the facility inventory instrument. The author also provided periodic in-person and remote instrument coaching and data quality assurance in-services. A trained clerk entered these data into the RedCap’s online database (Institute for Translational Health Sciences, 2007–2015). Accuracy of these data were confirmed using a standard double-entry technique where two assistants entered data from one quarter of the paper forms. Data clerks resolved any discrepancies between the two RedCap entries by reviewing the original paper forms. Thus, any discrepant REDCap entries were reconciled with the original paper records. The resulting validated database was used for analysis. RedCap data were exported to STATA for analysis (version 11.2, College Station, Texas, 1985–2009).

### Analysis

We described obstetric variables with standard descriptive statistics; point estimates for the availability of each resource are reported as percentages. Since the variables in this dataset had fewer than 100 observations, skewed distributions or did not follow symmetric Gaussian distribution, non-parametric descriptive and inferential statistics with two-sided tests significance were used for all analyses. Drop-offs in readiness between each stage were quantified with percentages. Central tendency was typically reported as the median. Means were used primarily for estimates of overall emergency readiness estimates in both models for two reasons: 1) the SRI methodology uses means and 2) since this measure is based on few observations the median would not capture the range of observations effectively or accurately. Variability was primarily summarized using absolute ranges since facilities varied widely in the obstetric resources available. Standard deviation (SD) and interquartile ranges (IQR) were selectively used as measures of variance when variation in the central tendency and range was of interest (for example using both metrics for the number of monthly deliveries illustrates wide variability in delivery volume by study site). Since global variability in urban-rural obstetric care is well-documented [3, 39, 54–56], we statistically quantified periurban/rural differences in the facility characteristics based on a facility’s rural status using Kenya MoH definitions.

To test differences between proportions, we used *Pearson’s chi-square test of independence* or *Fischer’s exact test* (for cell counts less than five). When comparing a variable’s distribution across unpaired categories, we used *Wilcoxon-ranked sum test* (for two categories) or Krus*kal-wallis’ h-test* (more than two categories). We used the *unmatched median test* to compare medians across two unpaired categories. The ‘signal function overestimate’ indicator is calculated by subtracting the novel cascade estimate of readiness from the standard signal function estimate of readiness (signal function estimate [–] clinical cascade estimate [=] readiness overestimate by signal function).

### Ethics

The activities and analysis of this nested study were all contained in the parent study approved by the University of Washington Institutional Review Board (43069) and the University of Nairobi Ethical Review Committee (P57/05/2012). The trial is registered in the PanAfrican Clinical Trials Registry (PACTR0121200045732, available from: http://www.pactr.org). Since the intervention targeted clinics and not individual clinical providers, prior to the clinic-level intervention, individual clinicians were verbally informed of the study and provided the opportunity to opt-out of the clinical training or assessments; no one opted out. Further, the MoH provided authorization to collect these data at the MoH facilities as part of the implementation trial. Consequently, the facility inventory data did not require individual informed consent.

## Results

### Facility characteristics

60% of facilities were rural and located within four Kakamega sub-counties: Khwisero, Butere, Matungu and Navakholo (S1 Table). 38% were open 24-hours and facilities conducted between 2–61 deliveries each month (S2 Table; mean = 10.50, median = 5.83, IQR = 4.67–13.50). In 24 hours, facilities had a median of 4 obstetric nursing staff on site (including licensed nurses, midwives and nurse auxiliaries) and 1 clinical officer/advanced practice clinician (S3 Table).

### Emergency obstetric resource availability

There was high variability in the availability of consumable supplies (range = 6.82–93.18%) and durable goods by facility (2.27–100%; S4 and S5 Tables). The presence of WHO first-line emergency medications varied by drug class [23, 51]. While most facilities stocked oxytocin (93.18%) and magnesium sulfate (72.73%), far fewer stocked ampicillin (4.55%) or hydralazine (9.09%; S6 Table). Except for a higher proportion of rural facilities with flashlights (72.00 versus 29.41%, Fisher’s exact p = 0.013), no statistically significant differences in resources availability were present based on a facility’s degree of urbanization (S3–S6 Tables).

### Signal function estimates of emergency readiness

**Fig 3:**
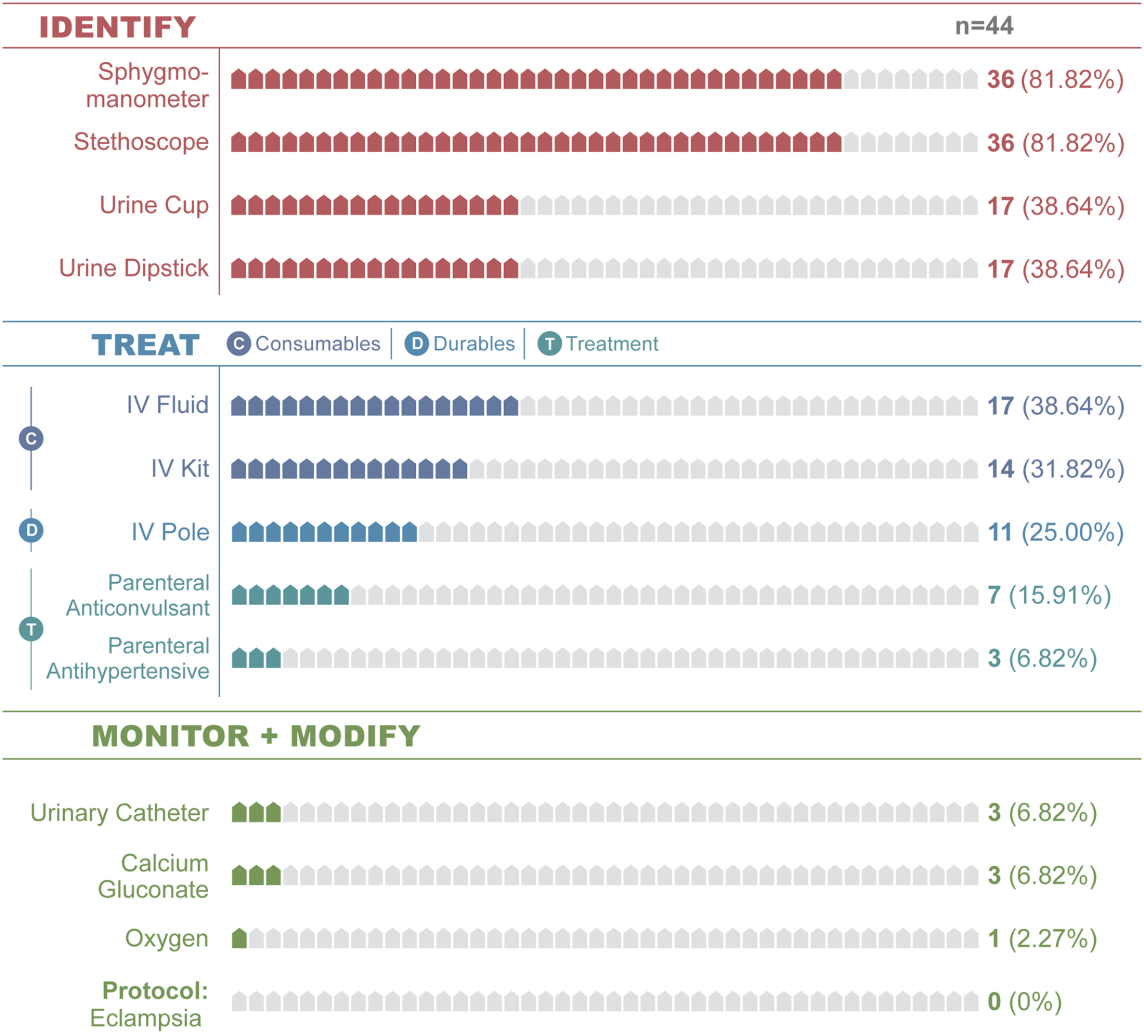
Hypertensive emergency clinical cascade.

Maternal signal function estimates of emergency readiness were quantified using nine standard tracers (S6 Table) [28, 33]. Readiness ranged from 75.45% (retained products of conception) to 93.18% (oxytocic; Tables 1–3; S1 and S2 Figs). However, readiness estimates based on tracers alone do not model how multiple resources are required sequentially or simultaneously for practical clinical management. For example, in hypertensive emergencies, facilities can treat the disorder only when all needed resources to identify and treat the emergency are simultaneously present (Fig 3). Of 44 facilities, 36 had sphygmomanometers and stethoscopes (88.12%). Of those, 17 (38.64%) had the testing supplies necessary to identify eclampsia or pre-eclampsia (urine collection cups and dipsticks to test for proteinuria). Although 72.73% stocked the magnesium sulfate tracer drug, a much lower proportion had resources to identify the emergency and administer the drug (38.64%, stage 2 emergency readiness; Figs 1 and 2). Far fewer also stocked the antihypertensive drug hydralazine that should be simultaneously administered with magnesium sulfate (6.82%, Table 1, Fig 3).

**Table 1:** Cascade emergency readiness stratified by medical signal function.

Clinical Cascade *(Signal Function)*	Cascade Stage	Item	%	n ^1^
**Manage Sepsis-Infection** *(Antibiotic)*	Identify	Thermometer	91.38%	41
		Stethoscope	91.38	41
		Sphygmomanometer	79.55	35
	Treat (Consumables)	IV Fluid ^2^	77.27	34
		IV Kit ^3^	70.45	31
	Treat (Durables)	IV Pole	52.27	23
	Treat (Treatments)	Parenteral Antibiotic-1 ^7^	47.73	21
		Parenteral Antibiotic-2 ^8^	40.91	18
	Monitor-Modify	Protocol: Infection	2.27	1
**Manage Hemorrhage** *(Oxytocic)*	Identify	Staff skill ^9^	100.00	44
	Treat (Consumables)	Gloves, aseptic	93.18	41
		IV Fluid ^2^	88.64	39
		IV Kit ^3^	81.82	36
	Treat (Durables)	IV Pole	59.09	26
		Refrigeration	56.82	25
	Treat (Treatments)	Parenteral Uterotonic ^10^	56.82	25
	Monitor-Modify	Sphygmomanometer	47.73	21
		Stethoscope	47.73	21
		Uterotonic, Secondary ^11^	47.73	21
		Urinary catheter	36.36	16
		Oxygen	6.82	3
		Protocol: Hemorrhage	2.27	1
**Manage Hypertensive Emergencies** *(Anticonvulsant)*	Identify	Sphygmomanometer	81.82	36
		Stethoscope	81.82	36
		Urine Cup	38.64	17
		Urine Dipstick	38.64	17
	Treat (Consumables)	IV Fluid ^2^	38.64	17
		IV Kit ^3^	31.82	14
	Treat (Durables)	IV Pole	25.00	11
	Treat (Treatments)	Parenteral Anticonvulsant ^12^	15.91	7
		Parenteral Antihypertensive ^13^	6.82	3
	Monitor-Modify	Urinary catheter	6.82	3
		Calcium Gluconate	6.82	3
		Oxygen	2.27	1
		Protocol: Eclampsia	0.00	0

^**(1)**^ Total sample n = 44 facilities;

^(2)^ Either normal saline (NS) or lactated ringer’s (LR);

^(3)^ IV cannula;

^(7)^ Parenteral ampicillin or parenteral penicillin (procaine, benzathine or cystalline);

^(8)^ Parentral gentamicin;

^(9)^ 100% staff skill for identifying the emergency disorder was assumed

^(10)^ oxytocin or misoprostol;

^(11)^ oxytocin, misoprostol or egometrine;

^(12)^ magnesium sulfate;

^(13)^ hydralazine

**Table 2:** Cascade emergency readiness stratified by manual signal function.

Clinical Cascade *(Signal Function)*	Cascade Step	Item	%	n ^1^
**Manage Retained Placenta** *(Manual removal of retained placenta)*	Identify	Staff Skill ^9^	100.00	44
		Light Source ^4^	77.27	34
	Treat (Consumables)	Gloves, Aseptic	72.73	32
		IV Fluid ^2^	68.18	30
		IV Kit ^3^	63.64	28
	Treat (Durables)	IV Pole	47.73	21
		Refrigeration	45.45	20
	Treat (Treatments)	Parenteral Uterotonic (oxytocin)	45.45	20
		Parenteral Sedative (diazepam)	43.18	19
		Parenteral Antibiotic-1 ^7^	38.64	17
	Monitor-Modify	Sphygmomanometer	31.82	14
		Stethoscope	31.82	14
		Uterotonic, Secondary ^11^	13.64	6
		Parenteral Antibiotic-2 ^8^	9.09	4
		Urinary catheter	9.09	4
		Protocol: Retained Placenta	0	0
		Protocol: Infection	0	0
		Protocol: Hemorrhage	0	0
**Manage Incomplete Abortion** *(Manual removal of retained products of conception)*	Identify	Speculum	88.64	39
		Light Source	72.73	32
	Treat (Consumables)	Gloves, Sterile	68.18	30
		IV Fluid ^2^	63.64	28
		IV Kit ^3^	59.09	26
	Treat (Durables)	Manual Vacuum Aspirator	29.55	13
		IV Pole	25.00	11
	Treat (Treatments)	Local Anesthetic (e.g., lidocaine)	18.18	8
		Parenteral Antibiotic-1 ^9^	18.18	8
	Monitor-Modify	Sphygmomanometer	18.18	8
		Stethoscope	18.18	8
		Refrigeration	18.18	8
		Uterotonic, Secondary ^11^	11.36	5
		Parenteral Antibiotic-2 ^8^	9.09	4
		Catheter, Urinary	9.09	4
		Protocol: Incomplete Abortion	2.27	1
		Protocol: Infection	0.00	0
		Protocol: Hemorrhage	0.00	0

^(1)^ n = 44 facilities;

^(2)^ normal saline (NS) or lactated ringer’s (LR);

^(3)^ IV cannula;

^(4)^ Functioning flashlight or functioning electric lights;

^(7)^ Parenteral ampicillin or parenteral penicillin (procaine, benzathine or crystalline);

^(8)^ Parenteral gentamicin;

^(9)^ 100% staff skill for identifying the emergency disorder assumed

^(11)^ Misoprostol or ergometrine

**Table 3:** Comparison of emergency readiness using clinical cascades and signal functions.

	Readiness Estimates by Model		
Clinical Cascade	Signal Functions	Clinical Cascades	Overestimated Readiness
*(Signal Function)*	% Readiness, Tracer Items	% Readiness, Stage 2	[Signal Functions (-) Cascade]
**Medical Treatments**			
**Manage Sepsis-Infection** *(Antibiotic)*	92.05%	40.91% ^1^	51.14
	(IV fluids, IV kit, ampicillin and/or penicillin, gentamicin)		
**Manage Hemorrhage** *(Oxytocic)*	93.18	56.82	36.36
	(Aseptic gloves, IV fluids, IV kit, oxytocin and/or misoprostol)		
**Manage Hypertensive Emergency** *(Anticonvulsant)*	86·36	6.82	79.54
	(IV fluid, IV kit, magnesium sulfate)		
*Mean Medical Readiness*	82.58	34.85	55.68
**Manual Procedures**			
**Manage Retained Placenta** *(Manual removal of retained placenta)*	86.74	38.64	48.10
	(Flashlight, IV fluids, IV kit, oxytocin, ampicillin and/or penicillin)		
**Manage Incomplete Abortion** *(Manual removal of retained products of conception)*	75.45	18.18	57.27
	(Flashlight, MVA, IV fluids, IV kit, ampicillin and/or penicillin)		
*Mean Manual Readiness*	81.10	38.64	42.64
**Mean Overall Readiness:**	86.76%	32.27%	54.48%
	*Signal Function Estimate*	*Cascade Estimate*	*% Overestimated by Signal Functions*

^(1)^ n = 44 facilities

### Clinical cascades

#### Overall readiness

**Fig 4:**
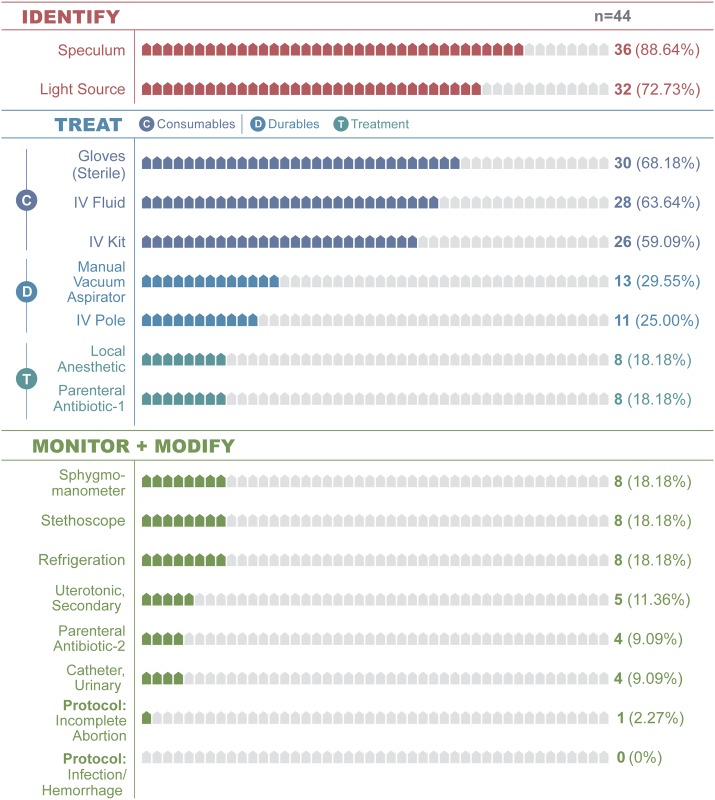
Incomplete abortion clinical cascade.

The signal function model overestimated basic obstetric emergency readiness by 54.48% (Table 3; Figs 1 and 2). It estimated a mean 80.94% readiness for all five emergencies (range = 75.45–93.18). However, a facility’s practical readiness as measured at the 2^nd^ stage of the cascade (emergency treatment) was substantively lower at 32.27% (range = 6.82–56.82%, Table 3; Fig 1). In addition, across clinical emergencies, signal functions consistently overestimate readiness by 33.03% with only moderate variance (SD = 18.94; Table 4). By signal function, the overall mean overestimates of readiness are: Antibiotic (51.14%), Oxytocic (36.36%), Anticonvulsant (79.54%), Placenta removal (48.10%) and Retained products removal (57.27%; Figs 2–4; S1–S3 Figs).

**Table 4:** Mean readiness loss by cascade and stage.

	Readiness Loss by Stage^1^			Readiness Loss by Cascade		
Loss by Clinical Cascade	1:	2:	3:	*Mean Loss Across 3 Cascade Stagese Stages*	*SD*	*Range*
	Identify	Treat	Monitor-Modify			
	--	--	--	*33*.*03*	*18*.*94*	*35*.*91*
**Sepsis-Infection**	*20*.*45%*	*38*.*64%*	*38*.*64%*	32.58	10.50	18.19
**Hemorrhage**	*0*.*00*	*43*.*18*	*54*.*55*	32.58	28.78	54.55
**Hypertensive Emergency**	*61*.*36*	*31*.*82*	*6*.*82*	33.33	27.30	54.54
**Retained Placenta**	*22*.*73*	*38*.*63*	*38*.*64*	33.33	9.18	15.91
**Incomplete Abortion**	*27*.*27*	*54*.*55*	*18*.*18*	33.33	18.93	36.37
**Overall Loss by Stage**						
*Mean Loss Across Cascade*	*22*.*31*	*33*.*71*	*23*.*72*			
*SD*	*25*.*18*	*20*.*39*	*22*.*55*	0.41		

^(1)^ n = 44 facilities

#### Readiness loss by cascade

There were notable differences in readiness loss along the cascades from identification of the disorder (stage 1) through monitoring-modifying therapy (stage 3). It varied least for the sepsis-infection (range = 18.19) and retained placenta cascades (range = 15.91) and most for hemorrhage and hypertensive emergencies (range = 54.55 and 61.36% respectively; Table 4; Fig 1; S4 Fig). There was also variability in when readiness was lost along the cascade of clinical care. For hemorrhage, the majority of readiness is lost in the monitor-modify stage (54.55%; Table 4; S1 Fig); in contrast, the hypertensive emergency cascade lost most readiness when identifying the disorder (61.36%; Table 4; Fig 3).

#### Readiness loss by stage

Across all five emergencies, the mean loss of readiness was 33.03% at each of three stages (Table 4). The loss across all five cascades was: 26.36% for emergency identification (stage 1), 41.36% for treatment (stage 2) and 31.37% for monitoring-modifying therapy (stage 3; Fig 2; S4 Fig). There was a profoundly consistent pattern of 33.03% overall readiness loss across emergencies and stages (SD = 0.41, Table 4) despite moderate variability in how loss occurred across these stages (SD across stages = 18.94).

## Discussion

Using the cascades and its family of novel indicators (practical emergency readiness, readiness loss by cascade, aggregate readiness loss across all cascades, readiness loss by stage) can provide multiple benefits for global program planners, health system policy makers, practice scholars and clinicians. The cascades offer a clinically-oriented yet population-health relevant, rapid, intuitive estimate of emergency readiness that is more precise than historic indicators. By quantifying where readiness loss occurs in the cascade of clinical care or by clinical emergency can more strategically guide morbidity and mortality-reducing interventions at the facility-, system- or global-level. Further, aggregate readiness loss across cascades is the first know indicator that can simultaneously quantifying a health system’s overall readiness and be used as a standardized comparison of readiness between systems, countries or regions.

Although broad facility inventories [28] and discrete tracer items are widely used globally, they are unable to accurately quantify a facility’s practical emergency readiness. The obstetric Service Readiness Index (SRI) partially rectifies these limitations by providing an overall summary measure of readiness for all six maternal signal functions [33]. However, neither inventories nor the readiness index provide a clinically relevant assessment of a facility’s practical ability to identify and manage common emergencies. To quantify practical readiness, cascades explicitly identify the interdependent relationship among resources [57, 58]. It models the relationship between identifying emergencies, treating them and then monitoring-modifying therapy based on clinical response (Tables 1 and 2; Figs 3 and 4). Although no known standards exist for measuring readiness for each maternal emergency signal function, the cascades provide emergency-specific readiness indicators (Tables 1, 2 and 4, Figs 3 and 4).

For reductions in facility-based obstetric mortality to occur on a global scale, a strategy-oriented approach to measuring readiness is needed. By adding 26 variables to existing inventories derived from the maternal signal functions [33], modeling resource interdependence and precisely defining variable ambiguities, the cascade achieves four goals. First, it identifies a 55% disparity between the high maternal signal function estimates of readiness (86.76%) and a facility’s actual readiness (32.27%; Table 4; Fig 1). Second, it summarizes a facility’s practical emergency readiness for each presenting clinical disorder (Figs 3 and 4, S1–S3 Figs). Third, it identifies points of readiness loss that predictably occur between identifying emergencies, treating them and monitoring-modifying therapy (Table 4, S4 Fig). Fourth, it offers a set of indicators for simultaneously measuring facility- and health system readiness. By defining treatment readiness as having all of the consumables, durables and treatments/drugs required to identify emergencies and give treatment, this model contains precise details about how and where readiness is lost (Tables 1, 2 and 3; Figs 1 and 2) and standardizes global estimates of readiness. Consequently, the cascade model could serve as a quantifiable, generalizable strategy for jointly assessing readiness and guiding strategic planning.

Since Stage 3 (monitor-modify therapy) is a benchmark for quality and not used to estimate readiness in the signal functions, it was not used to critique the existing maternal signal functions model. Including protocols to assess quality requires multiple protocols be used as tracers which complicates modeling and limits comparability with other studies since most existing facility readiness research does not include protocols (range = 2.27–11.36%, S6 Table). However, to maximize comprehensive emergency capacity, future work should model both the ability to provide essential emergency care (readiness indicator—stages 1–2) and to monitor-modify therapy (quality indicator—stage 3). These indicators can be combined into a single estimate of overall emergency capacity (stages 1–3 together measure quality which includes readiness).

Despite the existence of diverse facility inventory tools [28], little work has identified the variables most predictive of emergency readiness or produced guidance on how to strategically allocate emergency obstetric resources [13, 14, 59, 60]. The clinical cascade identifies discrete variables that can be used to measure and compare both readiness and quality by cascade, stage or specific resource with a minimal increase in data collection requirements compared to existing inventories based on the maternal signal functions (S7 and S8 Tables). The cascade approach also enables one to derive a summary of common resources required for multiple emergencies (S9 Table). By using this summary of common resources, one could strategically allocate resources that expand emergency readiness for multiple emergencies simultaneously.

These data are cross-sectional from 44 primary health centers in one region of east Africa. Since inventory data were collected for an intervention trial, some variables necessary for complete cascade modeling were not available (S7 and S8 Tables). However, any data gaps non-differentially affected both models so any potential misclassification bias in this study would simply underestimate the actual limitations of the maternal signal functions model [61, 62].

Ambiguities in how the maternal signal functions model defines tracer items may partially limit comparability between existing published estimates of global emergency readiness and this study. This suggests a model with more precise definitions is warranted to standardize global readiness estimates. The maternal signal functions poorly define some items (i.e., single parenteral antibiotic tracer does not differentiate between WHO stage 1, 2 and 3 condition-specific antibiotics) while some tracers actually contain multiple items (i.e., IV kits contains three discrete items). Ambiguous tracers were explicitly defined to maximize comparability between maternal signal function and cascade estimates of readiness. For example, although ampicillin is the stage 1 antibiotic tracer, few clinics stocked it (4.55%). Consequently, stage 1 readiness is modeled using any clinically indicated alternative drugs. The approach increased the number of clinics with stage 1 antibiotic readiness from 4.55 to 93.18% (Table 1; S2 Fig).

Future research could use the expanded cascades to compare the two estimates of readiness in additional contexts (S7 and S8 Tables). Process research could compare the cascade with others methods for measuring health system readiness using cost, cost-effectiveness or item response burden analyses. Outcome studies could test the cascade’s ability to predict facility-based morbidity, mortality, severe maternal outcomes or labor-related health outcomes for neonates [63–67]. The cascade logic could be applied to other obstetric situations [41, 68–79] including perinatal emergencies or routine clinical care for uncomplicated deliveries [35].

## Conclusions

Scholars and practitioners are calling for revisions in the signal functions in order to more precisely estimate global obstetric emergency readiness. This analysis demonstrates how maternal signal functions overestimate practical readiness by at least 55% and a consistent, predictable pattern of 33% capacity loss across all stages of emergency care exist in this context. This aggregate loss of readiness across cascades, stages and facilities provides the first known indicator for measuring, tracking and comparing health system emergency obstetric readiness. The cascades provide a quantitative, step-wise approach to clearly define basic obstetric emergency readiness. Practical, quantifiable assessments of readiness derived from the cascades can guide facility- and health system-level strategies for improving readiness and promoting maternal and neonatal survival at health facilities in low and middle income countries.

## Supporting information

S1 Fig Hemorrhage clinical cascade. (TIF)

S2 Fig Sepsis clinical cascade. (TIF)

S3 Fig Retained placenta clinical cascade. (TIF)

S4 Fig Mean emergency readiness loss by stage. (TIFF)

S1 Table Facility demographics. (DOCX)

S2 Table Facility accessibility and delivery volume. (DOCX)

S3 Table Human resources at facilities. (DOCX)

S4 Table Consumable supplies at facilities. (DOCX)

S5 Table Durable goods at facilities. (DOCX)

S6 Table Availability of signal function tracer items by type. (DOCX)

S7 Table Expanded clinical cascades: Medical treatments. (DOCX)

S8 Table Expanded clinical cascades: Manual procedures. (DOCX)

S9 Table Strategic resources for multiple basic emergencies. (DOCX)
