# Supplementary figures and images for: Beyond signal functions in global obstetric care: Using a clinical cascade to measure emergency obstetric readiness
Source: PLoS One. 2018 Feb 23;13(2):e0184252. doi: 10.1371/journal.pone.0184252 (PMC5825011; doi:10.1371/journal.pone.0184252)

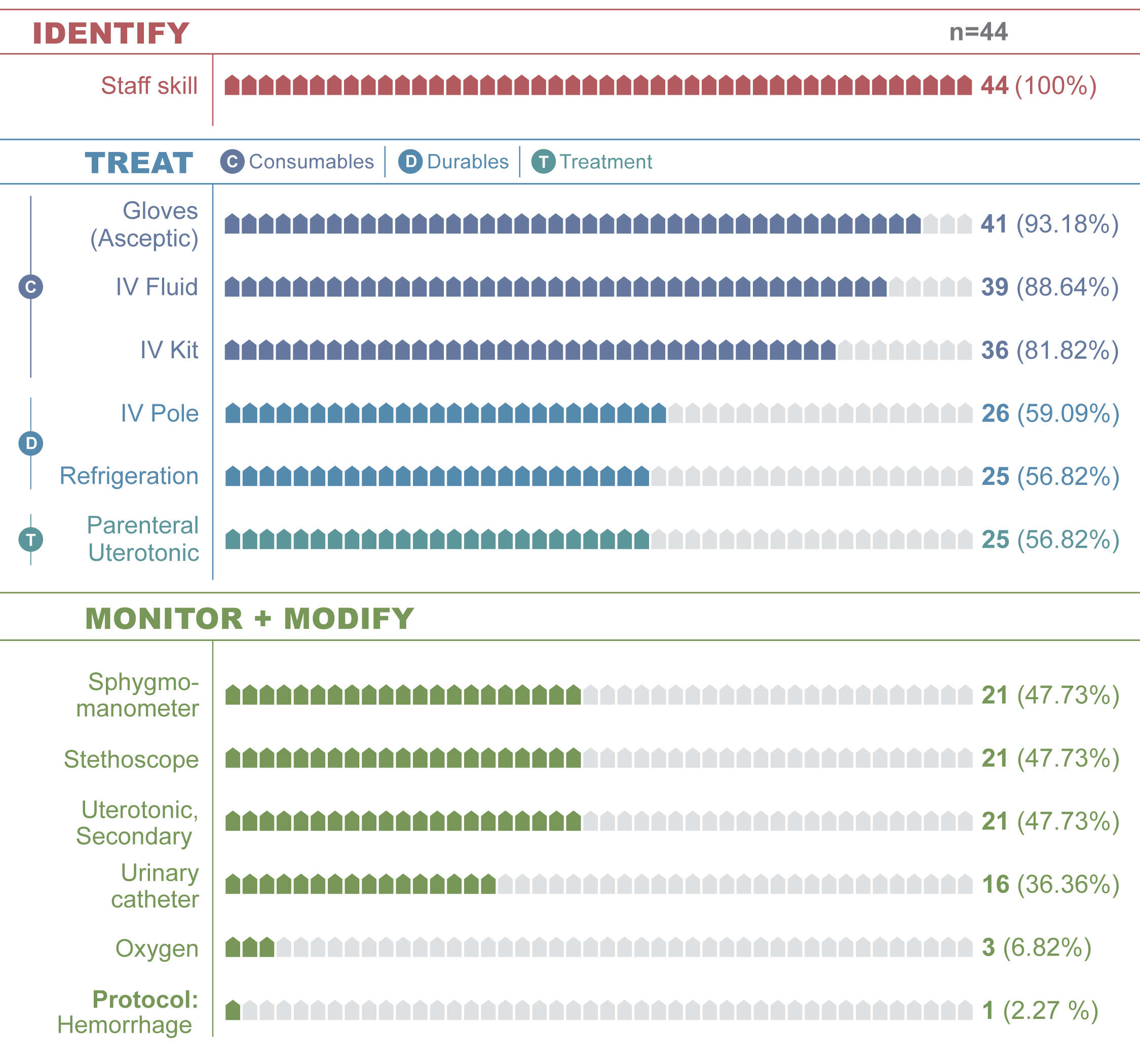

Supplement: S1 Fig — (TIF) [file pone.0184252.s001.tif]

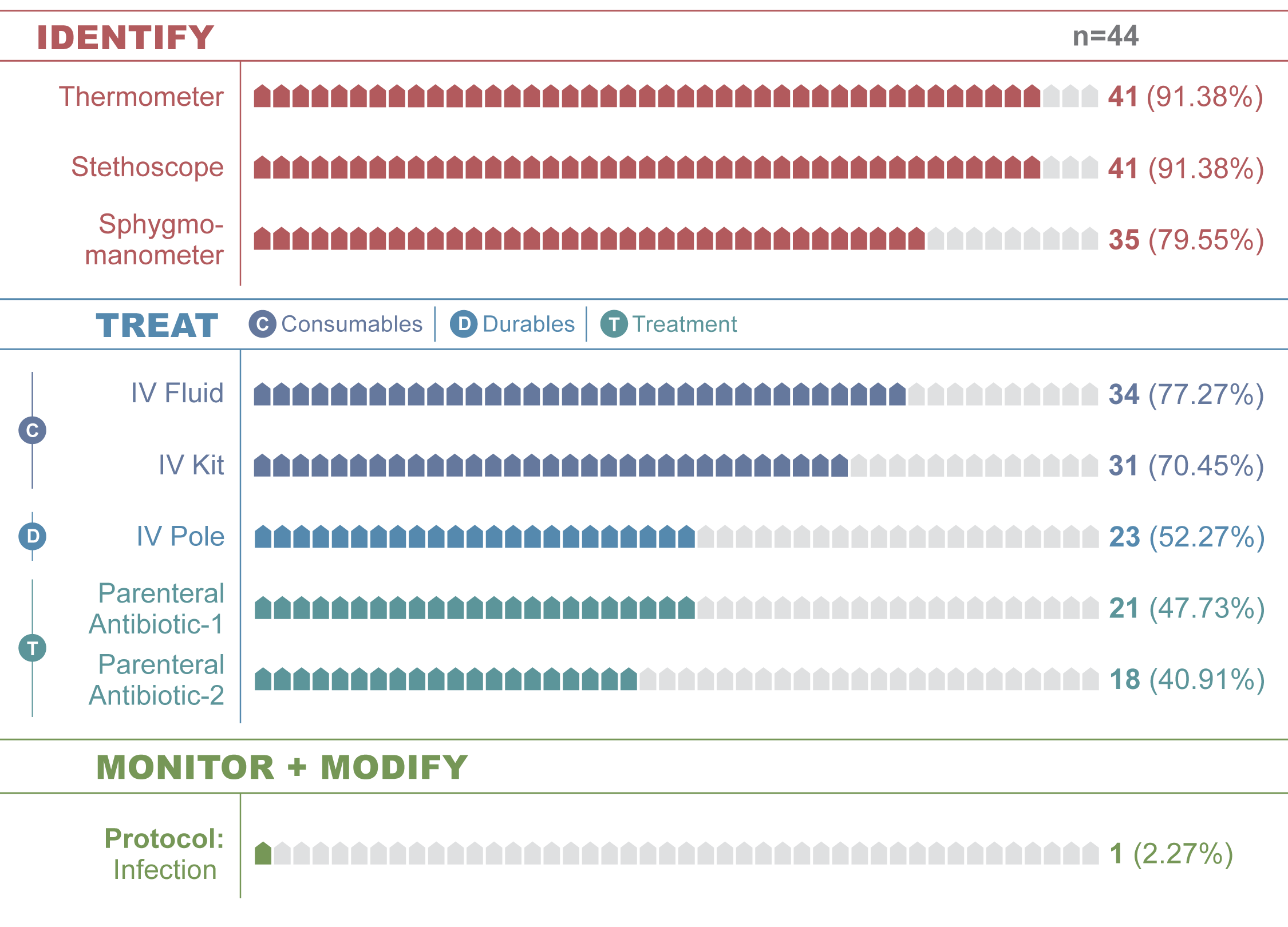

Supplement: S2 Fig — (TIF) [file pone.0184252.s002.tif]

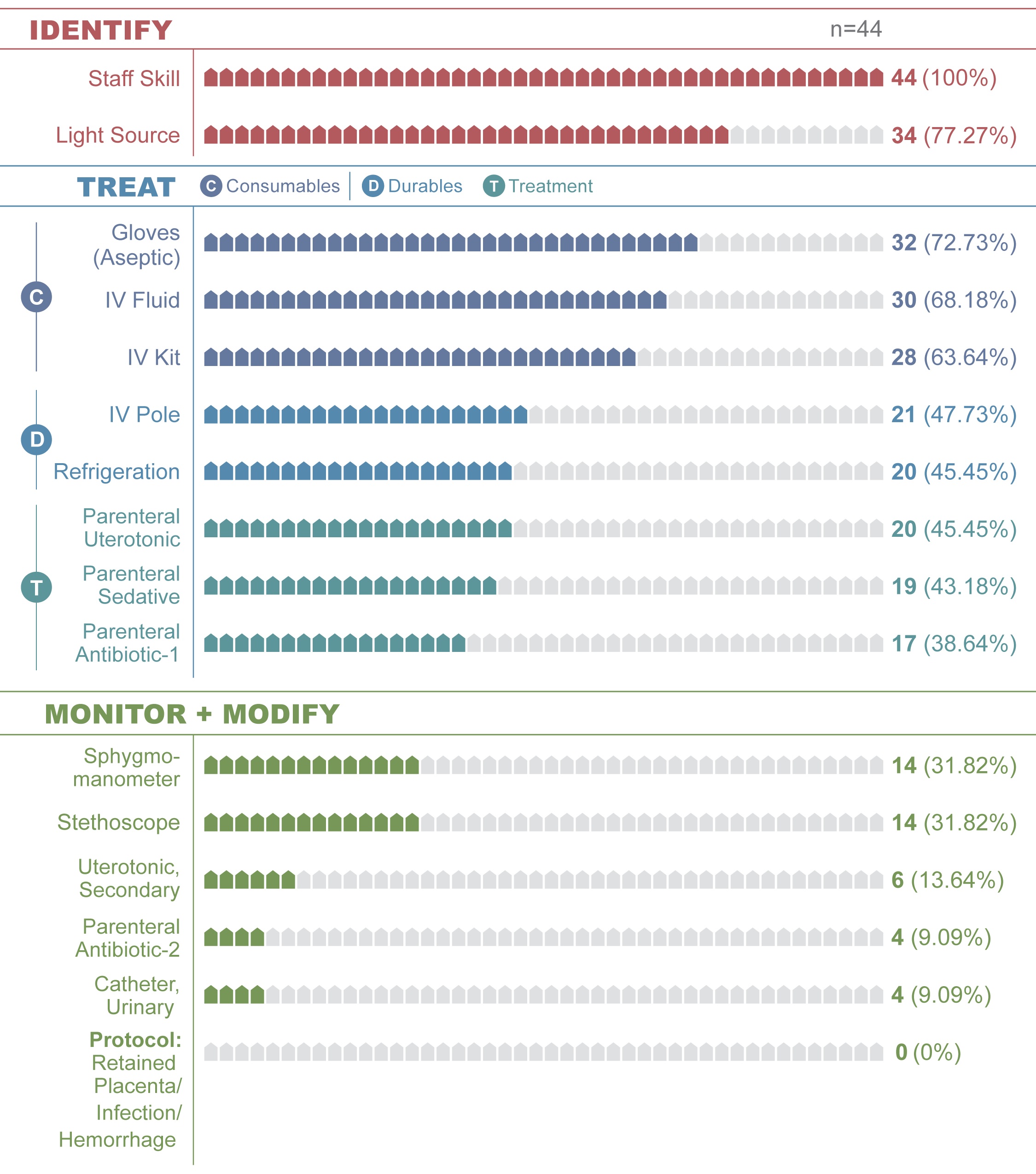

Supplement: S3 Fig — (TIF) [file pone.0184252.s003.tif]

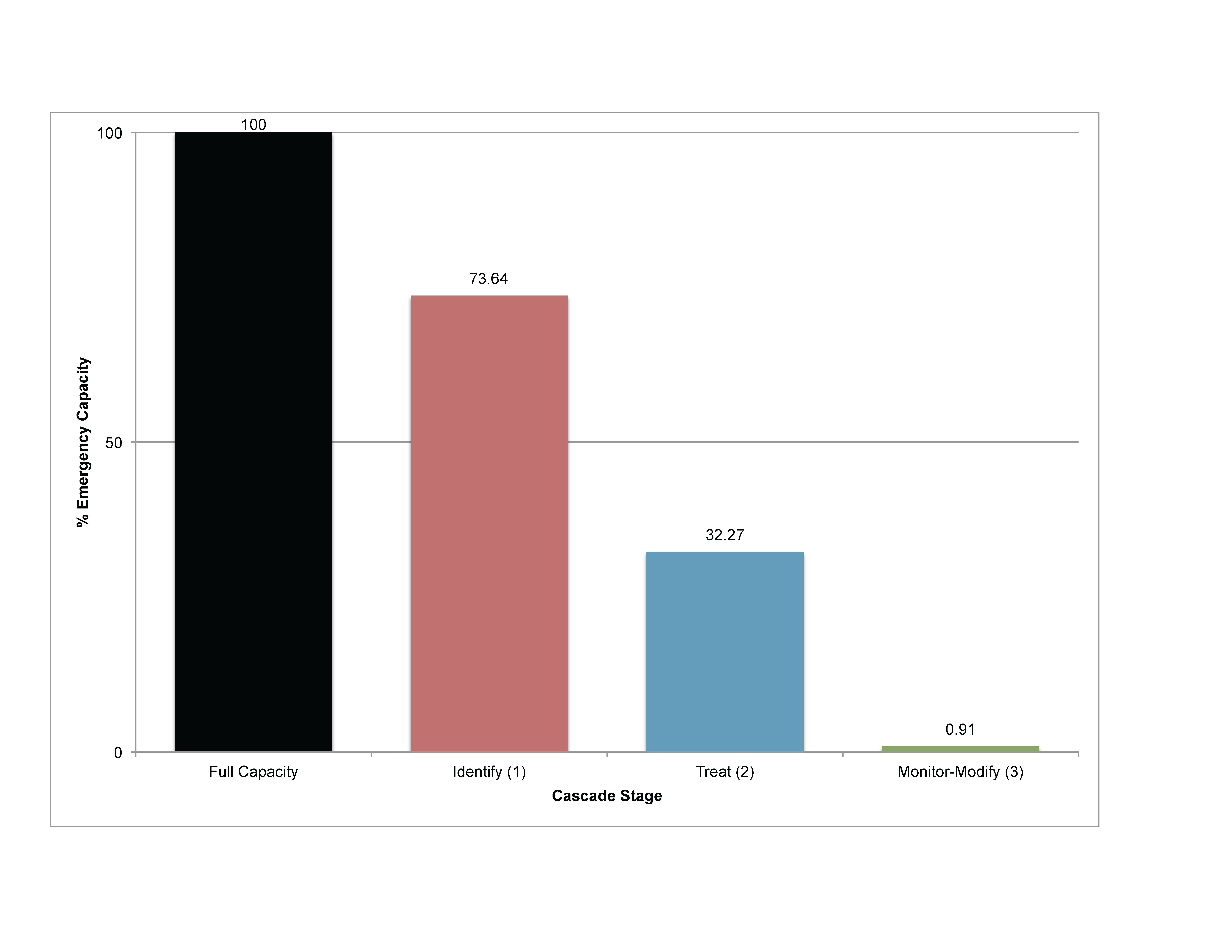

Supplement: S4 Fig — (TIFF) [file pone.0184252.s004.tiff]
