# Supplementary material for: Beyond signal functions in global obstetric care: Using a clinical cascade to measure emergency obstetric readiness
Source: PLoS One. 2018 Feb 23;13(2):e0184252. doi: 10.1371/journal.pone.0184252 (PMC5825011; doi:10.1371/journal.pone.0184252)
Supplement: S1 Table — (DOCX) [file pone.0184252.s005.docx]

**S1 Table: Facility Demographics**

|  | **Category** | **Sub-Category** | **%** | **n** ^1^ |
| --- | --- | --- | --- | --- |
| **Location** | *All Districts* | *All Facilities, Total* | *100.00%* | *n=44* |
|  | Peri-Urban Districts | Periurban Facilities, Total | 38.63% | 17 |
|  |  | Mumias | 20.45 | 9 |
|  |  | Kakamega Central | 18.18 | 8 |
|  | Rural Districts | Rural Facilities, Total | 61.36% | 27 |
|  |  | Khwisero | 20.45 | 9 |
|  |  | Butere | 20.45 | 9 |
|  |  | Matungu | 15.91 | 7 |
|  |  | Navakholo | 4.55 | 2 |
| **Facility Categorization** | KEPH Level  (Kenya Essential Package of Health) | 2 | 45.45 | 20 |
|  |  | 3 | 54.55 | 24 |
| (1) n=44 facilities | | | | |
