# Supplementary material for: Beyond signal functions in global obstetric care: Using a clinical cascade to measure emergency obstetric readiness
Source: PLoS One. 2018 Feb 23;13(2):e0184252. doi: 10.1371/journal.pone.0184252 (PMC5825011; doi:10.1371/journal.pone.0184252)
Supplement: S2 Table — (DOCX) [file pone.0184252.s006.docx]

**S2 Table: Facility Accessibility and Delivery Volume**

| **Category** | **Sub-Category** | **Median (%)** | **IQR (n)** ^1^ | **Periurban vs. Rural p-value** |
| --- | --- | --- | --- | --- |
| **24-Hour Access** | Yes | 38.64% | (17) | 0.718 ^d^ |
| **Delivery Volume by Facility** ^2^ | Yearly Deliveries | 70 | 50 – 175 | 0.412 ^a^ |
|  | Monthly Deliveries | 5.83 | 4.17 – 14.58 | 0.412 ^a^ |
| **Referral Facility Accessibility** | Distance  to nearest facility | 10 kilometers | 5 – 20 | 0.319 ^a^ |
|  | Time  by vehicle to nearest facility | 30 minutes | 15 - 35 | 0.118 ^a^ |
| (1) n=44 facilities; (2) The monthly and yearly estimates are based on extending the measured 6-month total volume from October 2012-April 2013 to the entire year total (yearly estimate multiplies measured 6-month total by 2) and to the monthly total (monthly estimate divides 6-month total deliveries by 6)·  (a)Wilcoxon rank sum test; (d) Unmatched median test | | | | |
