# Supplementary material for: Beyond signal functions in global obstetric care: Using a clinical cascade to measure emergency obstetric readiness
Source: PLoS One. 2018 Feb 23;13(2):e0184252. doi: 10.1371/journal.pone.0184252 (PMC5825011; doi:10.1371/journal.pone.0184252)
Supplement: S3 Table — (DOCX) [file pone.0184252.s007.docx]

**S3 Table: Human Resources at Facilities**

|  | **Profession** | **Sub-Category** | **Median (%)** | **IQR (n)** ^1^ | **Periurban vs. Rural p-value** |
| --- | --- | --- | --- | --- | --- |
| **Clinical Staff** | Obstetric Nursing Staff  (Auxiliary Nurses, Licensed Nurses and Midwives) | Any Nursing Staff | (100.0%) | (42) | -- |
|  |  | Nurses / Facility | 4 | 3-5 | 0.825 ^a^ |
|  |  | 24-Hours Presence | (45.45%) | (20) | 0.865 ^b^ |
|  | Clinical Officer  (Advanced Practice Clinician) | Any Clinical Officer | (47.73% ) | (21) | 0.242 ^b^ |
|  |  | Clinical Officers / Facility | 1 | 1-2 | 0.581 ^a^ |
|  | Medical Officer  (Family Physician) | Any Medical Officer | (2.27%) | (1) | 1.000 ^c^ |
| **Support Staff** | Laboratory | Any Laboratory Technologists | (63.64%) | (28) | 0.907 ^b^ |
|  |  | Lab Technologists / Facility | 1 | 1-1 | 0.304 ^a^ |
|  | Pharmacy | Any Pharmacy Staff | (18.18%) | (8) | 0.455 ^c^ |
|  |  | Pharmacy Staff / Facility | 1 | 1-1 | 0.083 ^a^ |
|  | Community Health Worker Relationships | Any Link with CHWs | (95.45%) | (42) | 1.000 ^c^ |
|  |  | CHWs / Facility | 21.5 | 13 - 35 | 0.959 ^a^ |
| (1) n=44 facilities; (a) Wilcoxon rank sum test (b) Pearson’s chi-Square test of independence; (c) Fischer’s exact test | | | | | |
