# Supplementary material for: Beyond signal functions in global obstetric care: Using a clinical cascade to measure emergency obstetric readiness
Source: PLoS One. 2018 Feb 23;13(2):e0184252. doi: 10.1371/journal.pone.0184252 (PMC5825011; doi:10.1371/journal.pone.0184252)
Supplement: S4 Table — (DOCX) [file pone.0184252.s008.docx]

**S4 Table: Consumable Supplies at Facilities**

|  | **Category** | **Item** | **%** | **n** ^1^ | **Periurban vs. Rural**  **p-value** |
| --- | --- | --- | --- | --- | --- |
| **General Consumables** | Testing | Urine collection cups | 50.00% | n=22 | 0.353 ^b^ |
|  |  | Urine dipsticks | 65.91 | 29 | 0.431 ^b^ |
|  |  | Glucostix | 47.73 | 21 | 0.490 ^b^ |
|  | Procedures | IV Cannulas | 90.91 | 40 | 0.624 ^b^ |
|  |  | Urinary Catheters | 77.27 | 34 | 0.624 ^b^ |
|  | Hygiene &  Wound Care | Cotton Dressings | 93.18 | 41 | 0.302 ^b^ |
|  |  | Soap | 93.18 | 41 | 0.845 ^b^ |
|  |  | Suture | 84.09 | 37 | 0.803 ^b^ |
|  | Personal  Protection Equipment | Gloves, Aseptic | 93.18 | 41 | 0.549 ^c^ |
|  |  | Gloves, Sterile | 88.64 | 39 | 1.000 ^c^ |
|  |  | Protective Clothing | 50.00 | 22 | 0.062 ^c^ |
| **Intravenous Fluids** | First Line | Normal saline (NS) | 88.64 | 39 | 0.634 ^c^ |
|  |  | Lactated ringers / hartman solution (LR) | 61.53 | 27 | 0.122 ^b^ |
|  | Second Line | Glucose with Saline | 6.82 | 3 | 0.549 ^c^ |
|  |  | 5% Glucose | 90.91 | 32 | 1.000 ^c^ |
|  |  | 10% Glucose | 15.91 | 7 | 0.402 ^c^ |
|  |  | 50% Glucose | 75.00 | 33 | 0.289 ^c^ |
|  |  | Sodium Bicarbonate | 34.09 | 15 | 0.963 ^b^ |
| (1) n=44 facilities; (b) Pearson’s chi-square test of independence; (c) Fischer’s exact test | | | | | |
