# Supplementary material for: Beyond signal functions in global obstetric care: Using a clinical cascade to measure emergency obstetric readiness
Source: PLoS One. 2018 Feb 23;13(2):e0184252. doi: 10.1371/journal.pone.0184252 (PMC5825011; doi:10.1371/journal.pone.0184252)
Supplement: S5 Table — (DOCX) [file pone.0184252.s009.docx]

**S5 Table: Durable Goods at Facilities**

|  | **Category** | **Item** | **%** | **n** ^1^ | **Periurban vs. Rural p-value** |
| --- | --- | --- | --- | --- | --- |
| **Delivery-Specific** | Reusable | Delivery Kit | 86.36 | 38 | 0.186 ^c^ |
|  |  | Towel | 20.45 | 9 | 0.445 ^c^ |
|  |  | Delivery Bed | 56.82 | 25 | 0.096 ^b^ |
|  |  | Manual Vacuum Aspirator | 43.18 | 19 | 0.831 ^b^ |
| **General** | Vital Signs | Thermometer | 93.18 | 41 | 0.845 ^b^ |
|  |  | Sphygmomanometer | 81.82 | 36 | 1.000 ^c^ |
|  |  | Stethoscope | 100.00 | 44 | -- |
|  |  | Fetoscope | 100.00 | 44 | -- |
|  |  | Doppler | 2·27 | 1 | 0.386 ^c^ |
|  | Physical Exam- Anthropometry | Measuring Tape | 15·91 | 7 | 0.689 ^c^ |
|  |  | MUAC Tape (Adult) |  |  | 0.505 ^c^ |
|  |  | Light Source ^4^ | 54.55 | 24 | 0.013 ^c^ |
|  |  | Speculum | 88.64 | 39 | 0.359 ^c^ |
|  | Testing | Glucometer | 45.45 | 20 | 0.651 ^b^ |
|  | Infrastructure | Clock | 52.27 | 23 | 0.944 ^b^ |
|  |  | Refrigeration^15^ | 95.35 | 41 | 0.549 ^b^ |
|  |  | Running water | 45.45 | 20 | 0.124 ^c^ |
|  |  | Electric Lights | 72.73 | 32 | 0.164 ^c^ |
|  |  | Oxygen | 6.82 | 3 | 0.272 ^c^ |
|  | Other  Instruments | Scissors | 75.00 | 33 | 0.075 ^c^ |
|  |  | Needle Holder | 65.91 | 29 | 0.894 ^b^ |
|  |  | Bedside Cart / Trolley | 52·27 | 23 | 0·074 ^b^ |
|  |  | IV Pole / Drip Stand | 72·73 | 32 | 0·739 ^c^ |
| (1) n=44 facilities; (4) Functioning flashlight or functioning electric lights; (15) Refrigerator or ice box present (b) Pearson’s chi-Square test of independence; (c) Fischer’s exact test | | | | | |
