# Supplementary material for: Beyond signal functions in global obstetric care: Using a clinical cascade to measure emergency obstetric readiness
Source: PLoS One. 2018 Feb 23;13(2):e0184252. doi: 10.1371/journal.pone.0184252 (PMC5825011; doi:10.1371/journal.pone.0184252)
Supplement: S6 Table — (DOCX) [file pone.0184252.s010.docx]

**S6 Table: Availability of Signal Function Tracer Items by Type**

| **Category** | **Type** | **Specific Tracer Item** | **%** | **n** ^1^ | **Periurban vs. Rural p-value** |
| --- | --- | --- | --- | --- | --- |
| **Consumables**  and **Durables** | Consumable Supplies | Gloves, Aseptic | 93.18% | n=41 | 0.549 ^c^ |
|  |  | IV Solution ^2^ | 95.45 | 42 | 0.515 ^c^ |
|  |  | IV Kit ^3^ | 90.91 | 40 | 0.624 ^b^ |
|  | Durable Goods and Infrastructure | Manual Vacuum Aspirator (MVA) | 43.18 | 19 | 0.831 ^c^ |
|  |  | Light source ^4^ | 77.27 | 34 | 0.030 ^c^ |
| **Drugs** | Uterotonic: First Line | Parenteral oxytocin | 93.18 | 41 | 0.272 ^c^ |
|  | Uterotonic: Alternatives | Oral misoprostol | 20.45 | 9 | 1.000 ^c^ |
|  |  | Parenteral ergometrine | 2.27 | 1 | 1.000 ^c^ |
|  |  | Oxytocin or misoprostol | 93.18 | 41 | 0.272 ^c^ |
|  |  | Any uterotonic ^5^ | 93.18 | 41 | 0.155 ^b^ |
|  | Antibiotic 1: First Line | Parenteral ampicillin | 4·55 | 2 | 1.000 ^c^ |
|  | Antibiotic 1: Alternative | Any parenteral penicillin^6^ | 93·18 | 41 | 1.000 ^c^ |
|  |  | Ampicillin or any parenteral penicillin | 93.18 | 41 | 1.000 ^c^ |
|  | Antibiotic 2: First Line | Parenteral gentamicin | 88.64 | 39 | 0.359 ^c^ |
|  | Anticonvulsant:  First Line | Parenteral magnesium sulfate | 72.73 | 32 | 0.343 ^b^ |
|  | Anticonvulsant: Alternative | Parenteral diazepam | 90.91 | 40 | 1.000 ^c^ |
| **Protocols - Algorithms** | Medical Treatments | Hemorrhage | 9.09 | 4 | 1.000 ^c^ |
|  |  | Eclampsia | 9·09 | 4 | 0.634 ^c^ |
|  |  | Infection-Sepsis | 4.55 | 2 | 1.000 ^c^ |
|  | Manual Procedures | Retained Placenta | 2.27 | 1 | 1.000 ^c^ |
|  |  | Retained Products | 9.09 | 4 | 1.000 ^c^ |
|  | General | Obstetric Emergency Manual | 11.36 | 5 | 1.000 ^c^ |
|  |  | Any Emergency Protocol or Emergency Manual Present | 38.64 | 17 | 0.784 ^b^ |
| (1) n=44 facilities; (2) Either normal saline (NS) or lactated ringer’s (LR); (3) IV cannula; (4) functional electric lights and electricity or flashlights (5) presence of one or more of the following parenteral drugs: oxytocin, misoprostol, ergotomine; (6) parenteral ampicillin or any parenteral penicillin alternative (benzathine, procaine or crystalline); (b) Pearson’s chi-square test of independence; (c) Fischer’s exact test | | | | | |
