# Supplementary material for: Beyond signal functions in global obstetric care: Using a clinical cascade to measure emergency obstetric readiness
Source: PLoS One. 2018 Feb 23;13(2):e0184252. doi: 10.1371/journal.pone.0184252 (PMC5825011; doi:10.1371/journal.pone.0184252)
Supplement: S7 Table — (DOCX) [file pone.0184252.s011.docx]

**S7 Table: Expanded Clinical Cascades: Medical Treatments**

| **Clinical Cascade**  *(Signal Function)* | **Cascade Stage** | **Item** |
| --- | --- | --- |
| **Manage Sepsis-Infection**  *(Antibiotic)* | Identify | Thermometer |
|  |  | Stethoscope |
|  |  | Sphygmomanometer |
|  | Treat (Consumables) | IV Cannula |
|  |  | IV Tubing |
|  |  | IV Fluid  (Normal saline or lactated ringer’s) |
|  |  | Syringe |
|  |  | Needle for syringe |
|  | Treat (Durables) | IV Pole |
|  | Treat (Treatments) | Parenteral antibiotic-1  (Ampicillin or penicillin alternative) |
|  |  | Parenteral antibioitic-2  (Gentamicin or cephalosporin alternative) |
|  |  | Parenteral Antibiotic-3  (Metronidazole) |
|  | Monitor-Modify | Protocol: Infection |
| **Manage Hemorrhage**  *(Oxytocic)* | Identify | Staff skill |
|  | Treat (Consumables) | Gloves, aseptic |
|  |  | Syringe |
|  |  | Needle for syringe |
|  |  | IV Cannula |
|  |  | IV Tubing |
|  |  | IV Fluid  (Normal saline or lactated Ringer’s) |
|  | Treat (Durables) | IV Pole |
|  |  | Electricity, Functional |
|  |  | Refrigerator, Functional |
|  | Treat (Treatments) | Parenteral oxytocin  (or oral misoprostol, parenteral tranexamic acid or carbetocene) |
|  | Monitor-Modify | Sphygmomanometer |
|  |  | Stethoscope |
|  |  | Secondary uterontonic (ergometrine or misoprostol) |
|  |  | Protocol: Hemorrhage |
| **Manage Hypertensive Emergency**  *(Anticonvulsant)* | Identify | Sphygmomanometer |
|  |  | Stethoscope |
|  |  | Urine Cup |
|  |  | Urine Dipstick |
|  | Treat (Consumables) | Syringe |
|  |  | Needle for Syringe |
|  |  | IV Tubing |
|  |  | IV Cannula |
|  |  | IV Fluid  (Normal saline or lactated Ringer’s) |
|  | Treat (Durables) | IV Pole |
|  | Treat (Treatments) | Parenteral Anticonvulsant  (magnesium sulfate or alternative such as diazepam ) |
|  |  | Parenteral Antihypertensive  (hydralazine or alternative) |
|  | Monitor-Modify | Urinary catheter |
|  |  | Calcium gluconate |
|  |  | Oxygen |
|  |  | Protocol: Eclampsia |
