# Supplementary material for: Beyond signal functions in global obstetric care: Using a clinical cascade to measure emergency obstetric readiness
Source: PLoS One. 2018 Feb 23;13(2):e0184252. doi: 10.1371/journal.pone.0184252 (PMC5825011; doi:10.1371/journal.pone.0184252)
Supplement: S8 Table — (DOCX) [file pone.0184252.s012.docx]

**S8 Table: Expanded Clinical Cascades: Manual Procedures**

| **­ Clinical Cascade**  *(Signal Function)* | **Cascade Stage** | **Item** |
| --- | --- | --- |
| **Manage Retained Placenta**  *(Manual removal of retained placenta)* | Identify | Staff Skill |
|  |  | Light Source  (Functional flashlight and battery or functional electricity and lights) |
|  |  | Urine Cup |
|  |  | Pregnancy Test |
|  | Treat (Consumables) | Gloves, Aseptic |
|  |  | Syringe |
|  |  | Needle for Syringe |
|  |  | IV Tubing |
|  |  | IV Cannula |
|  |  | IV Fluid (Normal saline or lactated ringer’s) |
|  | Treat (Durables) | IV Pole |
|  | Treat (Treatments) | Parenteral Oxytocin (or carbetocene) |
|  |  | Parenteral Sedative-Hypnotic  (Diazepam or alternative) |
|  |  | Parenteral Antibiotic-Step 1  (Ampicillin or penicillin alternative) |
|  |  | Parenteral analgesia  (Such as eemerol, roradol or alternative) |
|  | Monitor-Modify | Sphygmomanometer |
|  |  | Stethoscope |
|  |  | Uterotonic, non-oxytocin  (Misoprostol or ergometrine) |
|  |  | Parenteral Antibiotic-Step 2  (Gentamicin or cephalosporin alternative) |
|  |  | Catheter, Urinary |
|  |  | Protocol: Retained Placenta |
|  |  | Protocol: Infection |
|  |  | Protocol: Hemorrhage |
| **Manage**  **Incomplete Abortion**  *(Manual removal of retained products of conception)* | Identify | Speculum |
|  |  | Light Source  (Functional flashlight and battery or functional electricity and lights) |
|  | Treat (Consumable) | Gloves, Sterile |
|  |  | Syringe |
|  |  | Needle for Syringe |
|  |  | IV Tubing |
|  |  | IV Cannula |
|  |  | IV Fluid (normal saline or lactated ringer’s) |
|  |  | MVA cannula |
|  | Treat (Durable) | MVA device |
|  |  | IV pole |
|  | Treat (Treatment) | Local Anesthetic (such as lidocaine) |
|  |  | Parenteral Antibiotic-Step 1  (Ampicillin or penicillin alternative) |
|  | Monitor-Modify | Sphygmomanometer |
|  |  | Stethoscope |
|  |  | Electricity, functional |
|  |  | Refrigerator, functional |
|  |  | Uterotonic, Non-Oxytocin  (Misoprostol or ergometrine) |
|  |  | Parenteral Antibiotic-Step 2  (Gentamicin or cephalosporin alternative) |
|  |  | Catheter, Urinary |
|  |  | Protocol: Incomplete Abortion |
|  |  | Protocol: Infection |
|  |  | Protocol: Hemorrhage |
| **Manage Prolonged Labor**  *(Assisted Vaginal Delivery)* | Identify | Partograph, Blank |
|  |  | Writing instrument (pen or pencil) |
|  |  | Light Source  (Functional flashlight and battery or functional electricity and lights) |
|  | Treat (Consumable) | Gloves, Aseptic |
|  | Treat (Durable) | Assistive Device (Vacuum or Forceps) |
|  | Treat (Treatment) | None |
|  | Monitor-Modify | Suture material |
|  |  | Suture needle |
|  |  | Suture Forceps |
|  |  | Scissors |
|  |  | Syringe |
|  |  | Needle for syringe |
|  |  | Local Anesthetic (such as lidocaine) |
|  |  | Protocol: Assisted Vaginal Delivery |
|  |  | Protocol: Hemorrhage |
