# Supplementary material for: Beyond signal functions in global obstetric care: Using a clinical cascade to measure emergency obstetric readiness
Source: PLoS One. 2018 Feb 23;13(2):e0184252. doi: 10.1371/journal.pone.0184252 (PMC5825011; doi:10.1371/journal.pone.0184252)
Supplement: S9 Table — (DOCX) [file pone.0184252.s013.docx]

**S9 Table: Strategic Resources for Multiple Basic Emergencies**

|  |  | **Clinical Cascades Requiring Resource** | | |
| --- | --- | --- | --- | --- |
| **Category** | **Resource** | **n** | **%** | **Clinical Cascades** |
| **Identify Emergency**  or  **Monitor-Modify Treatment** | Sphygmomanometer | 5 | 83.3% | Sepsis-Infection, Hemorrhage, Hypertensive Emergency,  Retained Placenta, Incomplete Abortion |
|  | Stethoscope | 5 | 83.3 | Sepsis-Infection, Hemorrhage, Hypertensive Emergency,  Retained Placenta, Incomplete Abortion |
|  | Thermometer | 3 | 50.0 | Sepsis-Infection, Retained Placenta,  Incomplete Abortion |
|  | Urine collection cups | 2 | 33.3 | Hypertensive Emergency, Incomplete Abortion |
| **Consumables** | Syringe | 6 | 100.0 | Sepsis-Infection, Hemorrhage, Hypertensive Emergency,  Retained Placenta, Incomplete Abortion, Prolonged Labor |
|  | Needle for Syringe | 6 | 100.0 | Sepsis-Infection, Hemorrhage, Hypertensive Emergency,  Retained Placenta, Incomplete Abortion, Prolonged Labor |
|  | IV Fluid | 5 | 83.3 | Sepsis-Infection, Hemorrhage, Hypertensive Emergency,  Retained Placenta, Incomplete Abortion |
|  | IV Cannula | 5 | 83.3 | Sepsis-Infection, Hemorrhage, Hypertensive Emergency,  Retained Placenta, Incomplete Abortion |
|  | IV Tubing | 5 | 83.3 | Sepsis-Infection, Hemorrhage, Hypertensive Emergency,  Retained Placenta, Incomplete Abortion |
|  | Gloves, Aseptic | 3 | 50.0 | Hemorrhage, Retained Placenta, Prolonged Labor |
| **Durables** and **Infastructure** | Clean Water | 6 | 100.0 | Sepsis-Infection, Hemorrhage, Hypertensive Emergency,  Retained Placenta, Incomplete Abortion, Prolonged Labor |
|  | IV Pole | 5 | 83.3 | Sepsis-Infection, Hemorrhage, Hypertensive Emergency,  Retained Placenta, Incomplete Abortion |
|  | Electricity | 3 | 50.0 | Hemorrhage, Retained Placenta, Incomplete Abortion |
|  | Flashlight and Batteries  (or electric lights and electricity) | 3 | 50.0 | Retained Placenta, Incomplete Abortion, Prolonged Labor |
|  | Refrigerator with Electricity | 2 | 33.3 | Hemorrhage, Retained Placenta |
| **Treatment Drugs** | Antibiotic-2  (Gentamicin or cephalosporin class) | 4 | 66.7 | Sepsis-Infection, Hemorrhage, Retained Placenta, Incomplete Abortion |
|  | Uterotonic,  (Oxytocin or alternative) | 3 | 50.0 | Hemorrhage, Retained Placenta, Incomplete Abortion |
|  | Antibiotic-1  (Ampicillin or penicillin class) | 3 | 50.0 | Sepsis-Infection, Retained Placenta, Incomplete Abortion |
|  | Antibiotic-3  (Metronidazole) | 3 | 50.0 | Sepsis-Infection, Retained Placenta, Incomplete Abortion |
|  | Uterotonic, non-oxytocin | 2 | 33.3 | Retained Placenta, Incomplete Abortion |
|  | Local Anesthetic  (Lidocaine or alternative) | 2 | 33.3 | Incomplete Abortion, Prolonged Labor |
